# What does better look like in individuals with severe neurodevelopmental impairments? A qualitative descriptive study on SCN2A-related developmental and epileptic encephalopathy

**DOI:** 10.1007/s11136-023-03543-6

**Published:** 2023-12-08

**Authors:** Jenny Downs, Natasha N. Ludwig, Mary Wojnaroski, Jessica Keeley, Leah Schust Myers, Chere A. T. Chapman, JayEtta Hecker, Gabrielle Conecker, Anne T. Berg

**Affiliations:** 1 Telethon Kids Institute, Centre for Child Health Research, The University of Western Australia, PO Box 855, West Perth, WA 6872 Australia; 2 Curtin School of Allied Health, Curtin University, Perth, Australia; 3 Department of Neuropsychology/Psychiatry and Behavioral Sciences, Kennedy Krieger Institute/Johns Hopkins School of Medicine, Baltimore, MD USA; 4 Department of Psychiatry and Behavioral Health/Pediatrics, Nationwide Children’s Hospital/Ohio State University, Columbus, OH USA; 5 FamilieSCN2A Foundation, E. Longmeadow, MA USA; 6 Ardea Outcomes, Halifax, NS Canada; 7 DEEP Connections/SCN8A Alliance Wishes for Elliott, Washington, DC USA; 8 Department of Neurology, Northwestern Feinberg School of Medicine, Chicago, IL USA

**Keywords:** SCN2A, Developmental Epileptic encephalopathy, Qualitative, Clinical outcomes, Meaningful change

## Abstract

**Purpose:**

There are limited psychometric data on outcome measures for children with Developmental Epileptic Encephalopathies (DEEs), beyond measuring seizures, and no data to describe meaningful change. This study aimed to explore parent perceptions of important differences in functional abilities that would guide their participation in clinical trials.

**Methods:**

This was a descriptive qualitative study. Semi-structured one-on-one interviews were conducted with 10 families (15 parent participants) with a child with a SCN2A-DEE [8 male, median (range) age 7.5 (4.5–21)] years. Questions and probes sought to understand the child’s functioning across four domains: gross motor, fine motor, communication, and activities of daily living. Additional probing questions sought to identify the smallest differences in the child’s functioning for each domain that would be important to achieve, if enrolling in a traditional therapy clinical trial or in a gene therapy trial. Data were analyzed with directed content analysis.

**Results:**

Expressed meaningful differences appeared to describe smaller developmental steps for children with more limited developmental skills and more complex developmental steps for children with less limited skills and were different for different clinical trial scenarios. Individual meaningful changes were described as important for the child’s quality of life and to facilitate day-to-day caring.

**Conclusion:**

Meaningful change thresholds have not been evaluated in the DEE literature. This study was a preliminary qualitative approach to inform future studies that will aim to determine quantitative values of change, applicable to groups and within-person, to inform interpretation of specific clinical outcome assessments in individuals with a DEE.

**Supplementary Information:**

The online version of this article contains supplementary material available 10.1007/s11136-023-03543-6.

## Plain English summary

Developmental Epileptic Encephalopathies (DEE) are conditions that are often genetically caused with severe effects on child health and development. New medicines are being developed and tested in clinical trials and suitable measures are needed to assess and determine their benefits. It is important to understand what improvements are desired by individuals and their families to improve quality of life. Currently, there is no information on what changes in health and development are important for children with severe DEEs. We asked parents of children with a SCN2A-related DEE about important differences for their child’s functioning that would guide whether they would participate in clinical trials testing traditional therapies (lower risk) or disease-modifying gene therapies (higher risk). Parent-described meaningful changes varied by the severity of their child’s condition and the type of clinical trial. Future studies are needed to investigate meaningful change for groups of children and for individual children with a DEE, considering the amount of risk that the treatment could involve.

## Introduction

Developmental and epileptic encephalopathies (DEE) are rare conditions, typically characterized by early-onset refractory seizures, global developmental impairments, cortical visual impairment, movement disorders, and other associated medical symptoms, such as poor sleep and gastrointestinal dysfunction. There are a variety of associated behavioral disorders, such as autism spectrum disorders and attention-deficit/hyperactivity disorder (ADHD) [1, 2]. Advances in genetic testing techniques such as Next-Generation Sequencing (NGS) have redefined the genetic landscape of the epilepsies where hundreds of individually rare underlying genetic causes of DEEs have now been identified [3]. Most therapies target symptoms, such as seizures, not the underlying cause. Achieving control of symptoms and resolution of developmental impairments is likely to need new precision medicine therapies that target the genes, protein products, and molecular pathways associated with the genetic variant [4].

Beyond new treatments, best practice clinical trials need an understanding of the natural history of the condition, the distribution of genotypes and associations with phenotype, coordinated trial and clinical care networks, community readiness, and having fit-for-purpose outcome measures [1, 2, 5, 6]. A fit-for-purpose outcome measure needs to capture a relevant concept of interest and provide data that are reliable, valid, and responsive to meaningful patient change [7].

Measuring seizures is the traditional, go-to trial outcome for DEEs but seizures are not the only outcome of importance for the children and their families. For example, high-priority domains in the CDKL5 Deficiency Disorder relate to impairments in developmental and behavioral functioning, including communication [8]. Accordingly, non-seizure outcomes are receiving increasing attention as primary and secondary endpoints in clinical trials [9]. For children with severe impairments among the DEEs, there are limited validation data for commonly used measures of communication [10, 11], gross motor function [12], and quality of life (QOL) [13]. Further, the items within some tools may not be suitable to capture small increments in skills in children with severe impairments and therefore result in floor effects. For example, standardized domain scores of the Vineland Adaptive Behavior Scales-II (VABS-II) reflected performance approximately 3 SDs below the normative test average and dropped with age in SCN2A-DEE affected individuals in the Simons Foundation Autism Research Initiative project. By contrast, most raw scores increased with age and did not display the same floor effects. This is because standard scores may not capture the developmental gains that children with SCN2A-DEE are making which are occurring at a slower rate, in comparison to same-aged peers [14]. Alternative scoring methods to norm-referenced scores in measures such as the VABS could reduce floor effects and enable measurement of small yet meaningful changes in individuals with severe to profound impairments.

Beyond this, there are also no data to describe small differences in an outcome domain that would be considered important to patients. These are referred to as minimal clinically important differences (MCID) or minimal important differences (MID) [15; 16] and are critical to informing the definition of clinical trial endpoints. The MCID is usually determined using distribution or anchor-based approaches and is important to informing clinical trial study design and interpretation of results [15, 16]. To test the importance of a therapeutic effect in clinical trials, there is now a stated preference by the United States Food and Drug Administration to focus on within-person meaningful change rather than between-group differences [17–20]. Our clinical observations and consultations with affected families indicate that within-person meaningful change is identified as that which would enhance the quality of life of individuals with a DEE and of their parent caregivers, but this has not been investigated systematically. By extension, change that is important and meaningful to an individual could vary by factors within the clinical trial scenario, such as the risks of adverse events and burden of procedures. At present, we do not know how much of a difference in a domain is worthwhile for caregivers of severe to profoundly affected individuals with a DEE when considering clinical trial scenarios with different levels of risk. This information is a vital step for clinical trial readiness in DEEs for which novel therapies are now advancing toward clinical trials.

In this study, we conducted interviews with parents of children with SCN2A-DEE to explore their perceptions of important differences in their child’s functional abilities and activities of daily living. Specifically, we aimed to understand important differences that would guide their participation in different clinical trial risk scenarios, for traditional therapies (lower risk) and disease-modifying gene therapies (higher risk).

## Methods

### Study design

This was a descriptive qualitative study. Ethical approvals were granted by North Star Ethics Review Board, protocol NB200048 for the *SCN2A* Clinical Trials Readiness study (SCN2A CTRS) and protocol NB200063 for the current qualitative study. Parents provided informed electronic written consent to participate prior to the interview.

### Participants in pilot study

Parents who took part in this qualitative study were already participating in the *SCN2A* CTRS, which is a longitudinal study co-designed with parents to assess outcomes in their children that are life changing and ultimately important to parents. The *SCN2A* CTRS is a project of the FamilieSCN2A Foundation and aims to identify outcome measures appropriate for precision medicine clinical trials and it involves 65 families [21]. During the FamilieSCN2A Foundation annual family conference, in July 2022, the Inchstone Project (deepconnections.net/inchstone-project/) partnered with the *SCN2A* CTRS and the Foundation and conducted a pilot study to extend the type of outcome assessments that might be used in clinical trials in a subset of the CTRS cohort for which extensive outcome data were already available.

Convenience sampling was used because participants were invited to take part only if they were already participating in the SCN2A-CTRS and were attending the annual SCN2A Family meeting in-person with their child in Columbus, OH, July 28–29, 2022. While other assessments for the pilot were conducted on-site, the qualitative interviews were conducted remotely via internet conferencing after the family meeting. Parents from 10 families were recruited and no one declined to participate or withdrew from the study. The mother participated in all interviews and fathers in five. A second member of the investigator team joined three of the interviews. This sample size was considered sufficient for this pilot study to meet the criteria outlined by Malterud and colleagues [22] for achieving information power (i.e., considering the breadth of aim, phenomenon specificity, application of theory, dialogue quality, and analysis type).

Eight of the 10 children were male and their median (range) age was 7.5 (4.5–21) years. Data to describe comorbidities and expressive and receptive communication and gross motor and fine motor domain scores of the VABS-III, a measure of adaptive functioning, were extracted from the *SCN2A*-CTRS dataset. Most (n = 7) had been diagnosed with epilepsy and 4 had a gastrostomy insertion. All children had extremely low scores for each of the VABS-III domains (Table 1). The CTRS data included information about the child’s ability to walk (independent, with assistance, unable), communicate (uses some words, non-verbal communication), and grasp objects (picks up small objects, able to grasp large objects, unable to grasp objects) in broad categories. Four children had very severe impairments and could not walk independently, had minimal hand use, and were non-verbal. Six children had less severe impairments and were able to walk independently, had variable hand function but were non-verbal or minimally verbal. Summary data are presented in Table 1.

**Table 1:** Distribution of the child characteristics in the study sample and Vineland Adaptive Behavior Scores for communication and motor domains (*n* = 10)

Variable	Level	Number
Age group (years)	4–10	7
	> = 11	3
Biological sex	Male	8
	Female	2
Diagnosed with epilepsy	Yes	7
	No	3
Gastrostomy insertion	Yes	4
	No	6
Walking	Independent	6
	Walks with assistance	1
	Unable to walk	3
Use of hands	Picks up small objects	4
	Able to grasp large objects	3
	Unable to grasp objects	3
Communication	Uses some words	2
	Non-verbal communications	8
Toileting	Independence	3
Adaptive functioning	Domains	Standard V Scores Mean (SD)^#^
VABS-III	Expressive communication	1 (0)
	Receptive communication	1.6 (1.3)
	Gross motor skills	3.1 (3.6)
	Fine motor skills	2.6 (2.7)
	Personal*	1.6 (1.3)

^#^ Mean (SD) V scores for select subdomains, standardized to a mean of 15 and SD of 3

*Items describe self-sufficiency in daily living areas, such as eating, dressing, washing, hygiene, and health care

### Procedures

Semi-structured interviews were conducted to generate narratives describing the child’s functioning across four domains: gross motor, fine motor, communication, and activities of daily living. This was an initial exploratory study, and domains were selected because they are core functional components of the developmental disability in DEEs. Further, they map to adaptive behavior domains that are captured by well-known instruments (e.g., the VABS). Additional probing questions sought to identify the smallest differences in the child’s functioning in each of the domains that would be important to achieve for two clinical trial scenarios, if enrolling in a traditional therapy clinical trial and in a gene therapy trial. The risks of gene therapy trials were not described in detail because the interviews did not relate to a specific therapeutic trial, side effects would vary with the type and delivery of the gene therapy, and the gene therapy field is evolving. Rather, general information on risk was provided, for example, the gene therapy treatment could be associated with side effects, such as an inflammatory response [23]. Interviews took a concept elicitation approach. This method was considered the most appropriate as it allows for the exploration and definition of experiences from an individual’s perspective and can identify thresholds of meaningful change [17]. Further, this is a pilot study in an emerging area of research, therefore a theory-informed method would not have been appropriate [17]. Questions and probes sought to understand the child’s current skills and abilities, difficulties, consistency, and meaningful change that would inform their agreeing to participate in traditional therapy trials and in gene therapy trials. The interview schedule is presented in Online Resource 1.

Interviews were conducted by JD (PhD; female lead researcher) with extensive disability and qualitative research experience) in a single session with each family, with one or both parents depending on their preference. Participants had no prior relationship with JD and were informed of the study’s purpose and the researcher’s role and aims before interviews commenced. Field notes were taken throughout. Interviews were audio recorded and transcribed verbatim. The median (range) duration of the interviews was 48 (28–73) minutes.

## Data management and analysis

Interview transcripts were analyzed with a directed content analysis using NVivo. This type of analysis was considered the most appropriate to answer the research question because it requires the application of deductive categories [24]. Analysis was completed by both JD and JK. After becoming familiar with the transcripts, codes were extracted from the transcripts, informed by the principles of deductive qualitative content analysis. That is, codes describing functional abilities and meaningful change were identified for each functional domain. The transcripts were read and re-read and applicable text was coded to each functional domain (JD, JK). The categories and supporting data were presented to the parent participants for their feedback.

Trustworthiness—Strategies were taken during data collection and analysis to maintain credibility, transferability, dependability, and confirmability [25]. Review of findings with the investigator team and participating families was conducted to support the credibility of the analysis. Extensive and frequent reflective peer debriefing was used to limit investigator bias in coding when reviewing the transcriptions and both coders agreed on the final coding. For transferability of the data, rich descriptions of the data were provided. Dependability and confirmability were enhanced by the transparent and logical explanation of the steps and decisions made during the study. This was done using notes to document coding processes as an audit trail.

## Results

### Meaningful changes

**Fig. 1:**
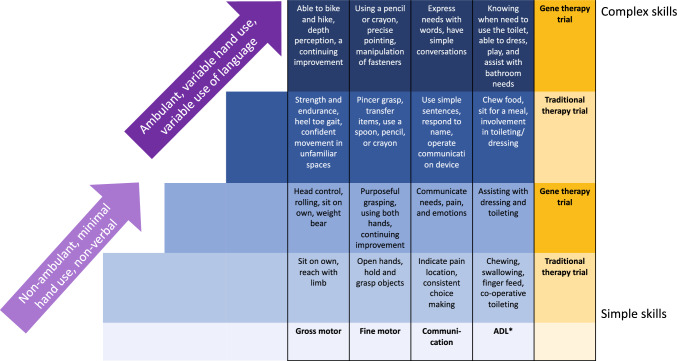
Summary of important meaningful changes for children with different levels of impairments and the two clinical trial scenarios. *ADL Activities of daily living. ^^^1 family did not have a meaningful gross motor change for traditional therapy trials and 2 families did not have a meaningful gross motor change for a gene therapy trial, because their child was confidently ambulant

Meaningful changes are presented in Fig. 1, for children with different levels of impairments and for the two clinical trial risk scenarios.

For traditional clinical trials, parents of children with more severe impairments described meaningful changes such as their child being able to sit on their own or take supported steps during transfers (gross motor), open their hand or volitionally grasp objects (fine motor), point to where it hurts or consistently make yes or no choices (communication), and chew and swallow food safely or cooperate with toileting (activities of daily living). Parents of children with more severe impairments described less complex meaningful changes for traditional clinical trials than those of children with less severe impairments. Parents of children with less severe impairments described meaningful differences such as their child being able to walk longer distances or confidence to move in unfamiliar spaces (gross motor), reduced repetitive hand movements or use a pencil (fine motor), use simple sentences (communication), and eat a whole meal or indicate when they need to pass urine (activities of daily living) (Fig. 1). The desired meaningful changes for traditional clinical trials were less complex than those for gene trials.

For gene therapy trials, parents of children with more severe impairments described meaningful differences, such as independent sitting for play (gross motor), purposeful grasping (fine motor), expressing their needs and emotions (communication), and participation in dressing (activities of daily living). Parents of children with more severe impairments described less complex meaningful changes for gene therapy trials than those of children with less severe impairments. Parents of children with less severe impairments described meaningful differences, such as being able to ride a bike (gross motor), use a pencil purposefully (fine motor), have simple conversations (communication), and manage toileting and dress independently (activities of daily living) (Fig. 1).

## Anticipated impacts of described meaningful changes

Many meaningful changes in skills were considered important for the child because use of these new skills could enable participation in activities that support good quality of life (Table 2). For example, some meaningful changes were described as having implications for the child’s emotional well-being (e.g., *“For her to convey her deeper feelings and experiences”*) and to support their socioemotional regulation *(*e.g., *“Being able to voice, ‘I don’t want to leave’ or ‘I'd like this instead’ would be so much better than lashing out”*). Other meaningful changes were described as important to improving capacity for participation in the community (e.g., “*More muscle strength for jumping and biking activities”*) and greater inclusion (e.g., *“She has sisters and being able to sit and play and interact with them. Being on the ground you get excluded a little bit just by default, even when the kids aren’t doing that. I think sitting would really make her feel more included”*). Some parents described how the meaningful changes could enhance the child’s educational progress (e.g., *“He is reading on a first-grade reading level—I would want him to be able to then take it a step further and hold a pencil, so that he can write out his words”*). Other meaningful changes could increase the child’s agency and independence (e.g., “*I would like to know what his preferences really are. A lot of times I’m just guessing and I'm giving him concrete choices*”).

**Table 2:** Summary of the implications of parent expressed meaningful changes for children and caregiving, classified by the levels of child impairment

Children	Gross motor	Fine motor	Communication	ADL
Implications for the child quality of life				
Non-ambulant, limited hand use, non-verbal	Participate in activities with others More independence	Point to where pain is so that it can be relieved Increased agency by communicating choices by pointing Greater ability to play Better engagement with the world	Identify pain so that it can be relieved Express emotions Increased agency by communicating choices	-
Ambulant, variable hand use, variable language use	Participate in leisure activities (e.g., hiking, bike riding, sports) More independence Increase mobility confidence generally and in different environments	Participate in sports Write own name	Increase child safety Increased agency by communicating choices Connect with others Exchange information with others	Improved ability to play Communicate choices Increase independence
Implications for caregiving				
Non-ambulant, limited hand use, non-verbal	Assist with transferring to reduce physical strain and avoid injury	Clarity in communication—understand choices	Clarity in communication—consistency Remove mystery—understanding what is wrong	Assist with tasks such as eating, dressing, and toileting results in less reliance on parents
Ambulant, variable hand use, variable language use	Participating equally in family activities and reduce activity restrictions Greater independence and less reliance on parents	Assist family with physical work, assist with dressing Clarity in communication—reducing child’s frustration	Keep child safe Improved mental health Communication clarity—reduce tantrums, Remove mystery—understanding what is wrong	Assist with tasks such as eating, dressing, and toileting results in less reliance on parents Understands child’s choices

**Fig. 2:**
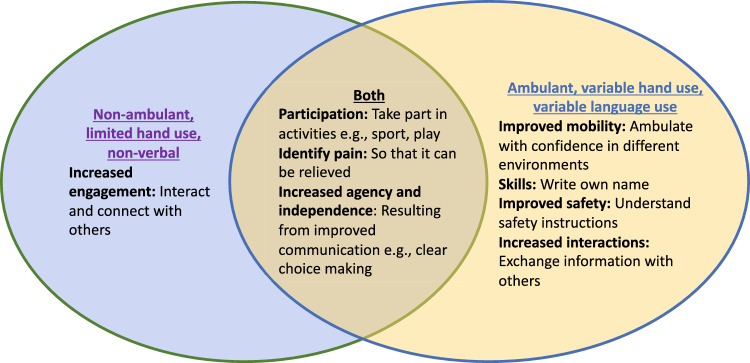
Implications of expressed meaningful changes for child quality of life across impairment levels and for impairment groups

There were similarities and differences across levels of impairment in the child’s functioning for the reasons that a change was identified as meaningful. For all abilities, skill gain was relevant to opportunities for activities (play or sport), identifying pain and increasing capacity for choice making. Some differences varied depending on the child’s impairments. For example, parents with more severely impaired children described how the meaningful differences could increase engagement with others, whereas parents with a less severely impaired child envisaged greater safety and confidence in the community (Fig. 2).

**Fig. 3:**
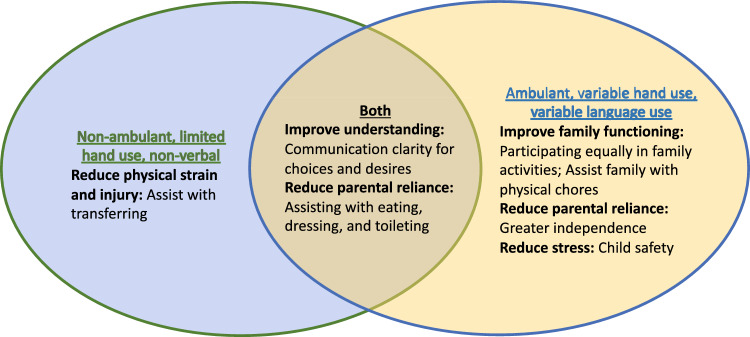
Implications of expressed meaningful changes for caregiving across impairment levels and for impairment groups

Other meaningful changes in skill were described as important for the provision of day-to-day care (Table 2). For children with severe impairments, new skills could enable easier transfers and reduce physical strain for caregivers (e.g., *“How long will we be able to care for him safely without injuring ourselves and then not being able to care for him”*). For children with less severe functioning, important gains in skill could support family functioning *by helping with chores*. For all abilities, skill gain was relevant to improving communication for parents to better gauge needs and wants and to reduce reliance on parents with greater participation in activities of daily living (Fig. 3).

## Discussion

Establishing meaningful change thresholds is critical for determining changes following treatments that are important to individuals and their families. Further, understanding meaningful change thresholds can determine whether measures are appropriately scaled to the patients’ levels of ability, can detect meaningful change in those abilities during the course of a trial, and whether the measure is fit-for-purpose and sensitive to change. This qualitative study was a preliminary approach to inform future studies that will aim to determine quantitative MCID and within-person meaningful change values for specific clinical outcome assessments in individuals with severe impairments, such as those with SCN2A and other DEEs.

We asked parents about the smallest differences in developmental skills that would be meaningful for their child to achieve, if enrolling in a traditional clinical trial or in a gene therapy trial. Expressed meaningful changes appeared to describe smaller developmental steps for children with severe to profound developmental functioning and more complex developmental steps for children with higher levels of skills. We recognize that beyond severity of impairments, meaningful changes could also vary by age and gender [26], and in DEEs, meaningful change could be influenced by comorbidities, such as refractory epilepsy or sleep difficulties. Parents considered the potential greater benefits and harms of gene therapy trials when considering important changes that they would want their child to achieve which were mostly “larger” than those described for traditional clinical trials [27]. This illustrates the notion that meaningful change is not an invariable characteristic of an outcome domain, but may depend, among other things, on the child’s baseline performance on the measurement instrument under study and the context within which the minimal importance is considered.

Despite the wide range of development skills described for the children in this study, all children had severe developmental impairments which is usual for children with a DEE [28]. As described earlier, using the Vineland Adaptive Behavior Scale for children with SCN2A-DEE [14], an observed floor effect in standardized scores would pose challenges to being able to (1) identify a meaningful change and (2) demonstrate whether a meaningful change was achieved. Fit-for-purpose outcome measures for multiple domains are needed for children who have complex and severe neurological impairments, such as DEEs. The work of the FamilieSCN2A Foundation Clinical Trial Readiness Study (CTRS) is an example where an education and advocacy program has partnered with researchers for rare genetic neurodevelopmental disorders [5, 29] including DEEs [30–32], working to achieve clinical trial readiness. Part of clinical trial readiness includes ensuring available scales are reliable and valid and have relevant items that are sensitive to small increments in skills and can measure change that is meaningful to the individual.

Parents described why the gains in skill were important, to enable a richer quality of life for the child or easier delivery of care. Many important changes were justified by their potential to improve how well the child lived, such as eating food during mealtimes, hugging a family member, increasing agency from clear choice making, and joining in with play opportunities and other activities in the community. Developmental skills do not equate to the child’s quality of life, but developmental skills allow engagement and participation in activities that are enjoyable and satisfying to the individual. The identified meaningful changes would likely have impacts across a range of domains consistent with the concept of quality of life [33] and is documented in multidimensional quality of life measures [34]. For families, improvement in the child’s quality of life was a key goal when considering undertaking new treatments, beyond improvement in disease symptoms.

Other meaningful changes were justified by their relationship with the challenges parents experienced when providing day-to-day care for the child. Parents with a child with a disability are vulnerable to poorer physical and mental well-being, particularly if the condition is clinically complex and severe [35]. It was hoped that meaningful changes in developmental skills would reduce reliance during care to protect parent and caregiver physical health into the future. Pain is a significant burden for many children with disability because of musculoskeletal or gastrointestinal comorbidities with implications for quality of life for the child [36]. Parents are experts in understanding their child’s pain but their evaluation remains uncertain and complex [37]. For caregivers, understanding the presence of pain and its distribution through better child communication would enable easier and less stressful care when supporting their child’s pain management.

There are several concepts related to the notion of meaningful change. First, change needs to be detected *reliably*. Test–retest reliability methods can be used to determine the Minimal Detectable Difference (MDD) and the standard error of measurement used to indicate the magnitude of difference that would be necessary to be 95% confident that the difference was greater than measurement error [38]. Second, the average of individual important changes in a group of patients needs to be understood for *utility*, to calculate sample sizes for clinical trials. The MCID refers to the difference in a scale score that is important for individuals, but averaged across a group of patients [15, 16], with the assumption that the scale can measure the meaningful change. Third, individual levels of change needs to be understood for what is *worthwhile*, *what is important for whom and when*, from patient and parent perspectives to enable trialists to interpret change in different trial scenarios [19]. Accordingly, all individuals would not need to achieve a change of at least the MCID or average group meaningful difference to be considered a responder [39]. For example, a child with severe impairments could achieve a small but meaningful change as described in the findings of this study but their change score could be smaller than a MCID value and they would be classified as a non-responder, yet they had achieved a meaningful change. All aspects in this family of change-related constructs need investigation for DEEs. Formal evaluations of MDD and MCID in groups and subgroups of patients are needed accompanied with mixed methods studies to investigate the range of change scores in individuals that represent meaningful change in different risk scenarios [27]. There is critical need to exploit opportunities in observational and intervention studies to evaluate the family of change-related concepts in DEEs.

This was a small convenience sample, which limits the transferability of the findings. The small sample was considered appropriate because this was a preliminary study conducted with parents attending an annual family conference. Nevertheless, we estimated that information power was achieved. Further, our findings are novel and generate needed preliminary data to inform progress in understanding meaningful change for children with DEEs. We acknowledge that larger studies are needed to determine meaningful differences for different ranges of scores and levels of impairment. We did not explore meaningful change in the direction of harm or reduction in scores and this is an important topic to investigate. This was a concept elicitation approach where parents discussed hypothetical change versus being able to reflect upon real changes that their child had experienced which is different information [17]. Therefore, future studies should also explore change in the context of real-world settings where actual change could be expected to occur or have occurred.

Non-seizure outcomes are important for health and quality of life in individuals with DEEs. Outcome measures that can measure meaningful differences are critical components for upcoming clinical trials. Meaningful change thresholds have not been evaluated in the DEE literature. Together with meeting reliability and validity criteria, data to understand the MDD, MCID, and within-person meaningful change are needed to achieve high-quality clinical trials where findings can be authentically applied to the population of interest. This study will inform future studies that aim to determine quantitative values for change, applicable to groups, and within-person, for specific clinical outcome assessments in individuals with a DEE.

### Supplementary Information

Below is the link to the electronic supplementary material.

Electronic supplementary material 1 (DOCX 17 kb)
