# Supplementary material for: What does better look like in individuals with severe neurodevelopmental impairments? A qualitative descriptive study on SCN2A-related developmental and epileptic encephalopathy
Source: Qual Life Res. 2023 Dec 8;33(2):519–28. doi: 10.1007/s11136-023-03543-6 (PMC10850209; doi:10.1007/s11136-023-03543-6)
Supplement: Supplementary file 1 — Electronic supplementary material 1 (DOCX 17 kb) [file 11136_2023_3543_MOESM1_ESM.docx]

**Appendix 1**

**POST-CONFERENCE INTERVIEW SCHEDULE – MINIMAL CLINICAL IMPORTANT CHANGES**

**INTRODUCTION TO THE TOPIC**

Thank you for joining this study and for sharing your time today.

This interview is about [child’s name] day-to-day functioning. We are interested to talk about changes that you feel would be important for them, for example, change that could occur with new medicines or treatments. More specifically, we will talk about motor, hand function, communication, and activities of daily living such as eating and dressing.

We hope to hear examples or stories that illustrate your child functioning.

*Do you have your copy of the interview questions in front of you?* So, we will start with the questions.

**DOMAIN QUESTIONS**

Gross motor skills

Can you tell me about how [child’s name] moves?

1. Prompts to explore head control, sitting, changing position, standing, walking, climbing and running
2. Does your child use their skills consistently?
3. [Individual Educational Plan goal if gross motor]

Let’s think generally about everything you have described about your child’s gross motor skills.

**If you going into a clinical trial that led to fewer symptoms, what improvement in [XXXX’s] gross motor skills would you want? What would the smallest change be that would be important?**

**If there was a new gene therapy treatment that had potential for large benefits for your child’ gross motor skills but bigger risks, what is the smallest change would make it worth your while to join the trial?**

Hand function

Can you tell me about how [child’s name] manages activities with their hands?

1. Prompts to explore grasping, playing with toys, drawing and writing, precision tasks such unlocking a door
2. Does your child use their skills consistently?
3. [Individual Educational Plan goal if hand function]

Let’s think generally about everything you have described about your child’s hand function.

**If you going into a clinical trial that led to fewer symptoms, what improvement in [XXXX’s] hand function skills would you want? What would the smallest change be that would be important?**

**If there was a new gene therapy treatment that had potential for large benefits for your child’ gross motor skills but bigger risks, what is the smallest change would make it worth your while to join the trial?**

Communication – expressive and receptive

Can you tell me about how [child’s name] communicates?

1. Prompts to explore noises, body language (facial expressions, eye-blinking, etc), reaching and pointing, single words, word approximations, phrases, sentences, yes/no cards, other alternative and augmented devices
2. Prompts to explore understanding requests and making choices
3. Does your child use their skills consistently?
4. [Individual Educational Plan goal if communication]

Let’s think generally about everything you have described about your child’s communication.

**If you going into a clinical trial that led to fewer symptoms, what improvement in [XXXX’s] communication skills would you want? What would the smallest change be that would be important?**

**If there was a new gene therapy treatment that had potential for large benefits for your child’ gross motor skills but bigger risks, what is the smallest change would make it worth your while to join the trial?**

Activities of daily living

Can you tell me how [child’s name] functions in daily activities?

1. Prompts to explore eating, dressing, toileting, managing transport
2. Does your child use their skills consistently?

Let’s think generally about everything you have described about your child’s independence in daily life.

**If you going into a clinical trial that led to fewer symptoms, what improvement in [XXXX’s] ability to do daily living skills would you want? What would the smallest change be that would be important?**

**If there was a new gene therapy treatment that had potential for large benefits for your child’ gross motor skills but bigger risks, what is the smallest change would make it worth your while to join the trial?**

General questions

Of the areas that we have discussed (gross motor, hand function, communication, independence etc),

1. Which has the most impact on [child’s name]?
2. Which has the most impact on you and your family?

Considering your child’s health and functional abilities overall,

1. Which problems have the biggest impact on how [child’s name] lives?
2. Which have the biggest impact on how you and your family live?

Your stories have told us so much about [child’s name]. Are there any other thoughts that you would like to share?

**Probing questions**

How do you know that this would be important?

What would I see or hear if I was there?

Why did you think that is?

**CONCLUSION AND THANK YOU**
